# Imaging single chiral nanoparticles in turbid media using circular-polarization optical coherence microscopy

**DOI:** 10.1038/srep04979

**Published:** 2014-05-15

**Authors:** Pengfei Zhang, Kalpesh Mehta, Shakil Rehman, Nanguang Chen

**Affiliations:** 1 Optical Bioimaging Lab, Department of Bioengineering, National University of Singapore, 7 Engineering Drive 1, Singapore 117576; 2 NUS Graduate School for Integrative Sciences and Engineering, National University of Singapore, 28 Medical Drive, Singapore 117456; 3 BioSystems and Micromechanics IRG, Singapore MIT Alliance for Research and Technology, 1 Create Way, Singapore 138602; 4 These authors contributed equally to this work.

## Abstract

Optical coherence tomography (OCT) is a widely used structural imaging method. However, it has limited use in molecular imaging due to the lack of an effective contrast mechanism. Gold nanoparticles have been widely used as molecular probes for optical microcopy based on Surface Plasmon Resonance (SPR). Unfortunately, the SPR enhanced backscattering from nanoparticles is still relatively weak compared with the background signal from microscopic structures in biological tissues when imaged with OCT. Consequently, it is extremely challenging to perform OCT imaging of conventional nanoparticles in thick tissues with sensitivity comparable to that of fluorescence imaging. We have discovered and demonstrated a novel approach towards remarkable contrast enhancement, which is achieved by the use of a circular-polarization optical coherence microscopy system and 3-dimensional chiral nanostructures as contrast agents. By detecting the circular intensity differential depolarization (CIDD), we successfully acquired high quality images of single chiral nanoparticles underneath a 1-mm-thick tissue -mimicking phantom.

Fluorescence microscopy has been an indispensible tool in modern biomedical sciences due to attributes that are not readily available in other contrast modes, such as absorption and scattering, with traditional light microscopy. The application of a wide range of fluorescent dyes has made it possible to identify sub-cellular components with a high degree of specificity^1^,^2^,^3^. In fact, the fluorescence microscope has become adequately sensitive to track single molecules^4^,^5^,^6^. However, there are some limitations when applying fluorescence microscopy to in vivo imaging of biological tissues. The most significant problem is the imaging depth. Confocal fluorescence microscopes are limited to about 250 microns in depth. Multi-photon fluorescence images can be obtained from a depth greater than 500 microns, depending on the laser power and tissue optical properties. Another well-known problem of fluorescence microscopy is photobleaching, an irreversible process of photochemical destruction of fluorescence dyes due to the light exposure necessary to excite them. Photobleaching is especially undesirable in time-lapse microscopy and quantitative analysis.

Optical coherence tomography (OCT) has evolved as a powerful optical imaging modality to obtain tomographic images with moderate spatial resolution (10–20 μm) but rather large imaging depth (2–3 *mm*) in biological tissues^7^. While a range of medical applications for OCT have been explored, ophthalmic imaging is so far the most successful one^8^,^9^. OCT is typically used to visualize layered structures in a tissue, which results in strong reflection even from a large depth. Cellular resolution imaging has become possible since optical coherence microscopy (OCM) was developed for better spatial resolution (~2 μm)^10^,^11^. However, even OCM has very limited application in basic biological research due to its low sensitivity and specificity in identifying sub-cellular organelles. While OCT and OCM use back-reflection and backscattering as the contrast mechanism, the scattering cross-section of microscopic particles drops rapidly with a decreasing dimension. For example, the Rayleigh cross section is proportional to the sixth power of the sphere diameter. Consequently, the scattering signal from tiny organelles can be easily overwhelmed by reflection from layered or large-scale tissue structures.

While the intrinsic sub-cellular OCT/OCM signal is very weak, plasmonic nanostructures have attracted much attention from researchers because of the surface Plasmon enhanced backscattering around the resonance wavelength. Gold nanoparticles (including nanoshells and nanorods) are believed to be the best candidates for OCT contrast agents. Gold nanoparticles, conjugated to various biomolecules, have been used with OCT to visualize molecular concentrations in thick biological tissues^12^,^13^,^14^. Nevertheless, the surface plasmon resonance enhancement may be far from adequate in deep tissue imaging, where the background signal is dominating. An extremely high concentration of nanoparticles may be required in order to make the targeting molecules or organelles visible.

Several groups have investigated various approaches to develop molecule specific contrast agents for OCT. Oldenburg *et al.* reported a technique to image magnetic nanoparticles in vivo^15^,^16^. A modulated magnetic field is used to induce a magnetomotive force and motion of magnetic nanoparticles, which results in detectable intensity differences in OCT images. Photothermal response of gold nanorods has been explored as another molecular OCT contrast. Photothermal OCT is able to detect and separate the absorbing target from the scattering background based on the temperature dependent refractive index change around the nanorods^13^. However, repeated heating in live cells may disrupt the normal biological processes and cause structural damage.

In previous work we demonstrated effective signal to background improvement by using the circular depolarization signal from asymmetrical nanoparticles^17^. We also had proposed to use the polarization dependent response of chiral nanoparticles as an effective background rejection mechanism^18^. Most biological tissues do not have a noticeable chiral response in the back-scattered direction in the near infrared wavelength range, which means that they have extremely weak (if any) circular dichroism and almost identical response to left-circularly and right-circularly polarized illumination^19^. In our previous work a 3D plasmonic chiral nanostructure (PCN), which consists of two nanorods separated by a small distance and forming a 45-degree angle, was numerically investigated using the finite difference time domain method (FDTD). A strong differential OCT signal between the left- and right-circular polarized illumination conditions was predicted based on ensemble averaging over random orientation of PCN's.

Here we report the experimental demonstration of the ultra-high sensitivity of detecting PCN's in a tissue phantom using OCM. Arrays of PCN's were fabricated using an E-beam method and imaged with a polarization OCM setup. The imaging results proved that it is possible to image individual plasmonic chiral nanoparticles in a highly scattering tissue phantom. Another array of nanorods was also fabricated to compare the achievable contrast with those of PCN's. It is evident that PCN, combined with a new chirality parameter, provides the best enhancement in signal to background ratio. We expect that such an ultra-sensitive contrast mechanism will significantly boost the utilization of OCT/OCM in basic life science research as well as medical diagnoses.

## Results

### Circular Polarization OCM (CP-OCM) set-up

**Figure 1:**
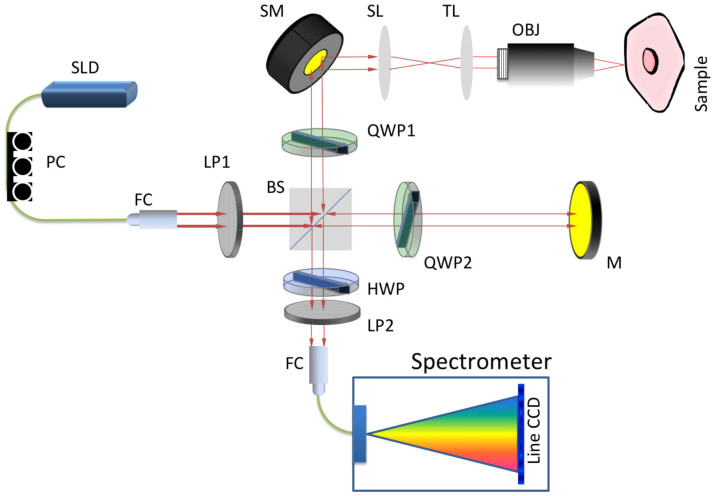
Schematic of CP-OCM Setup. Red lines represent free space optical paths and green lines represent fiber guided optical paths. SLD: Super-luminescence laser diode; FC: fiber coupler/collimator. LP: linear polarizer; BS: beam splitter; QWP: quarter-wave plate; M: mirror; SM: scanning mirror; SL: scanning lens; TL: tube lens; OBJ: objective; HWP: half-wave plate.

A Bioptigen spectral domain OCT system was modified to be circular-polarization sensitive. While most reported polarization OCT systems employ two detectors for simultaneous measurement of co- and cross-polarized components^20^, the Bioptigen system only has a single line camera for spectral domain measurement. As a result, co- and cross-polarized images have to be obtained sequentially using our CP-OCM system, which is schematically illustrated in Figure 1.

The illumination beam is generated by a super-luminescence laser diode (SLD), which has center wavelength of 840 nm and a bandwidth of 50 nm. The collimated beam is linearly polarized using a Glen-Thompson prism before entering a free space polarization independent beam splitter (50:50). The sample beam is converted to either a left- or right-circularly polarized beam by the use of an achromatic quarter-wave plate (QWP1). Lateral scanning is achieved by a 2-dimensional scanning mirror combined with a scan lens and a tube lens. The reference beam polarization is manipulated by a second quarter-wave plate (QWP2) and is reflected by a mirror back to the beam splitter. A near infrared objective (40×, 0.6NA) is used to tightly focus the sample beam into a sample. The backscatteed/backrefleced signal is collected by the same objective and is mixed with the reference beam at the beam splitter. A half-wave plate (HWP) is combined with another linear polarization (LP2) to selectively pass the co- or cross-polarized components in the mixed beam, which is convoyed to a spectrometer to generate a spectral domain interference pattern. The captured interference signal is then transferred to a PC (not shown in the figure), which performs Fourier transform and image rendering.

Directly obtained from the CP-OCM are four polarization sensitive images, *A_LC_*, *A_LX_,* *A_RC_*, and *A_RX_*, and the corresponding circular depolarized and co-polarized intensities 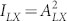, 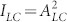, 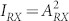, and 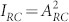. The subscriptions *L* and *R* respectively refer to left- and right-circularly polarized illumination, while *C* and *X* corresponds to co- and cross-polarized detection, respectively. The total backscattering intensity in response to left-circular-polarized illumination *I_L_* = *I_LC_* + *I_LX_* and the total backscattering intensity in response to right-circular-polarized illumination *I_R_* = *I_RC_* + *I_RX_* are generally used to calculate circular intensity differential scattering (CIDS) defined as: 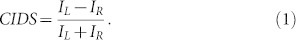
It is desirable that *I_LC_* and *I_RC_* cancel each other so that the difference between *I_LX_* and *I_RX_* stands out. In OCT imaging of a thick tissue, however, both *I_LC_* and *I_RC_* could be dominated by contributions from reflective layers and/or other scatterers surrounding the chiral nanostructure. As a result, the difference between *I_LC_* and *I_RC_*, subjecting to noises and the imbalance between different polarization channels, could overwhelm *I_LX_* and *I_RX_*. We have discovered a better chirality parameter for detecting the existence of PCN's. It is related to the depolarization ratios *CDR_L_* = *I_LX_*/*I_LC_* and *CDR_R_* = *I_RX_*/*I_RC_*. The circular intensity differential depolarization (CIDD), defined as, 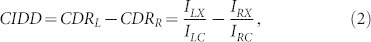
does not contain the difference between *I_LC_* and *I_RC_*. As one will see in the imaging results, CIDD provides a much better contrast than CIDS.

### Plasmonic Chiral Nanostructure (PCN) fabrication

**Figure 2:**
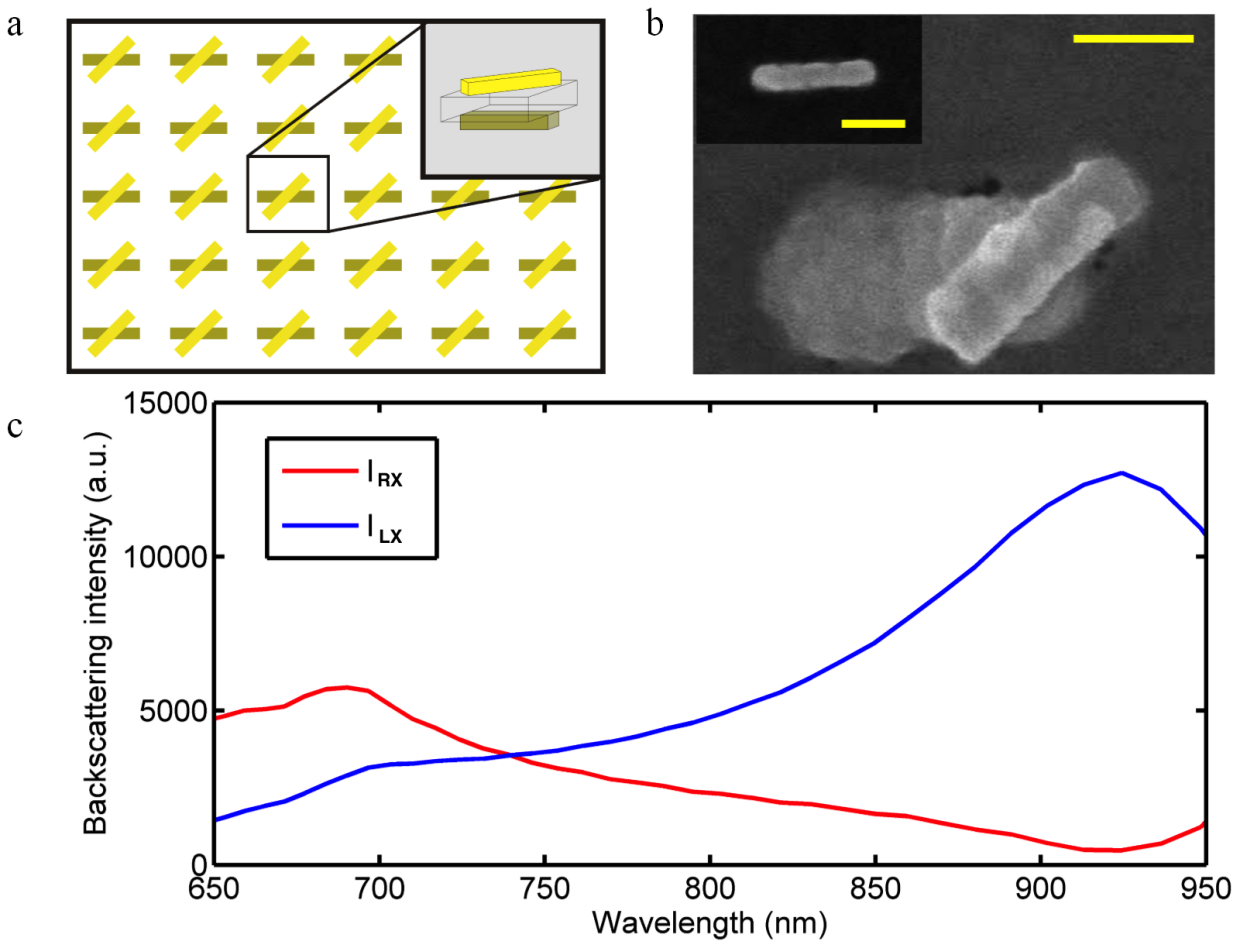
PCN array. (a) Geometry of a Plasmonic chiral nanoparticle array (inset: 3D sketch of an individual L-PCN) (b) SEM image of a single L-PCN (inset: SEM image of the bottom nanorod before the dielectric layer was deposited). Scale bars: 100 nm. (c) FDTD simulated backscattering (depolarization component) intensities for LCP (blue) and RCP (red) incident beams, respectively, from the L-PCN.

For experimental demonstration three nanostructure arrays, which consist of left handed PCN (L-PCN), right handed PCN (R-PCN), and nanorods (NR), respectively, were fabricated on separate silicon dioxide substrates using an established E-beam lithography protocol^21^. The arrangement of L-PCN array is shown in Figure 2a. The R-PCN and NR arrays use the same spatial arrangement. The lateral spacing between two neighboring particles is 10 μm and there are 50 × 50 particles in each array. The size of all nanorods is 175 nm × 50 nm × 50 nm, while in L-PCN and R-PCN the top and bottom NRs are separated by a 50 nm thick SiO_2_ dielectric layer.

Figure 2b shows the SEM image of a fabricated L-PCN. As the bottom layer of gold nanorods is below a 50-nm-thick SiO_2_ dielectric layer, the nanorods in this layer appear wider than their actual dimensions due to the limited penetration depth of SEM. The inset figure shows the actual size and shape of nanorods before dielectric layer deposition.

### Tissue phantom

**Figure 3:**
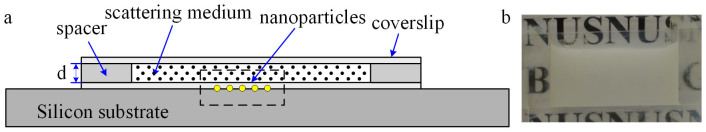
Tissue phantom. (a) Schematic and (b) photo of the tissue phantom.

A tissue phantom was designed and fabricated to emulate the situation where nanoparticles are embedded deep inside the biological tissue. Figure 3a shows the phantom placed on top of a substrate carrying nanoparticles. The phantom is a 1-mm-thick cell filled with Lipofundin of various concentrations to mimic the scattering and absorption properties of human soft tissue^22^,^23^. A typical concentration of 1% was used in our imaging experiments. Strong scattering of visible light by such a phantom can be seen in Figure 3b.

### Imaging results

**Figure 4:**
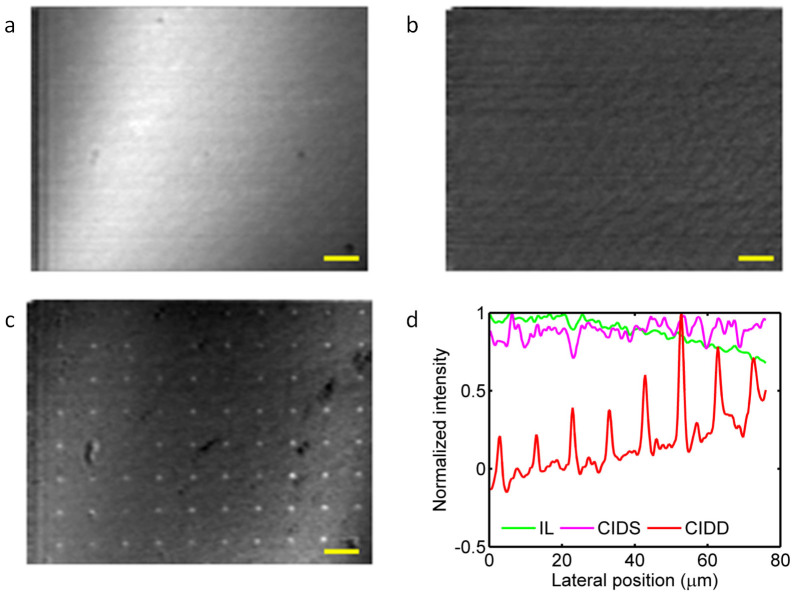
Enface images of the L-PCN array. (a) Conventional OCT image. (b) CIDS image. (c) CIDD image. Scale bars: 10 μm. (d) Normalized signal intensities along a line across several PCN's.

The L-PCN's on substrate were at first imaged directly using the CP-OCM system and the en-face images are shown in Figure 4. The L-PCN's can hardly be seen in the conventional OCM image (represented by *I_LC_* in Figure 4a) as the reflection from the substrate resulted in a very strong background. However, it was unexpected that even the CIDS image (Figure 4b) does not provide adequate background rejection for visualizing the PCN's. In contrast, the particles are clearly visible in the CIDD image (Figure 4c). Normalized line profiles are compared in Figure 4d. The conventional OCM signal (green) is essentially featureless while the CIDS signal (purple) appears quite noisy. The CIDD signal (red), on the other hand, provides a remarkable signal to background ratio.

**Figure 5:**
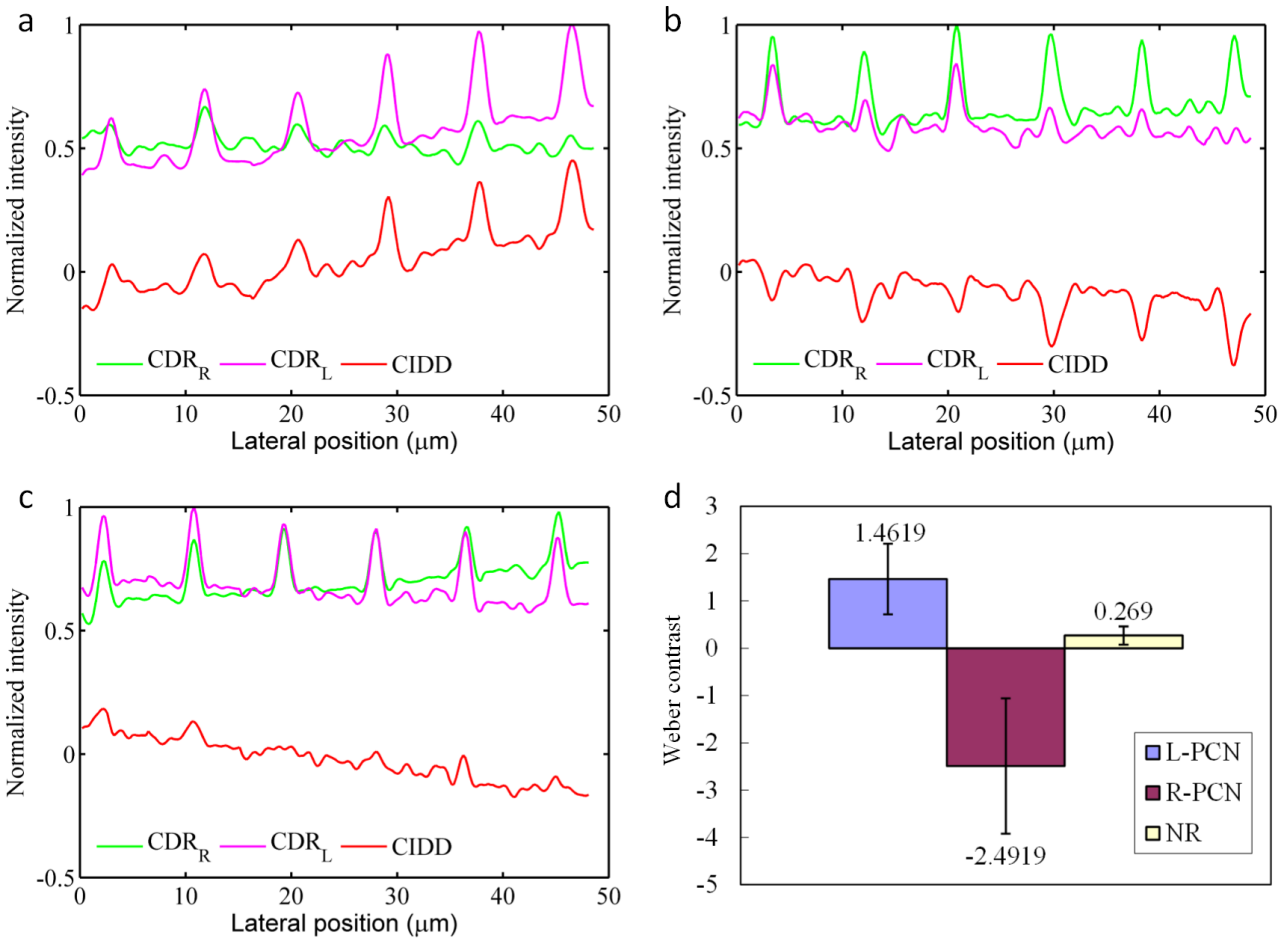
L-PCN, R-PCN, and NR comparison. Normalized Line profile of CDR for LCP illumination (Purple line), RCP illumination (Green line) and CIDD (Red line) for (a) L-PCN, (b) R-PCN and (c) NRs, respectively. (d) Weber contrast for L-PCN, R-PCN and NR.

To further quantify and prove the origin of contrast enhancement, CDR and CIDD imaging results for L-PCN, R-PCN and NRs are compared. Figure 5a shows the normalized line profiles from L-PCN's CDR_L_ (Purple line), CDR_R_ (Green line), and CIDD (Red line) images. It can be seen that CDR_L_ provides reasonably good background rejection for L-PCN's. The peaks in CDR_R_, however, appear much smaller due to the chirality of the nanostructures. CIDD uses the differential depolarization response to further reduce the background significantly. The depolarization responses are reversed in case of R-PCN (Figure 5b), and the CIDD becomes negative in PCN regions. Depolarized backscattering intensities are almost equally strong for NR's (Figure 5c), resulting in a nearly zero CIDD. This is compatible with the fact that NR's are achiral.

To quantify the contrast enhancement, the signed Weber contrast^24^, 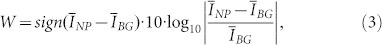
can be calculated from the images. Here 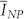 is the average image intensity in the nanoparticle region and 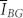 is the average image intensity in the surrounding background region. The sign of this parameter is used to indicate the handedness of nanostructures. Figure 5d compares the Weber contrasts obtained from the CIDD images of L-PCN, R-PCN, and NR. It is evident that only PCN's can generate reliable chiral responses. The difference between the contrasts of L-PCN and R-PCN can be explained by the non-ideal polarization response of the CP-OCM system, in which imperfect optical components and misalignment may lead to unwanted depolarization.

**Figure 6:**
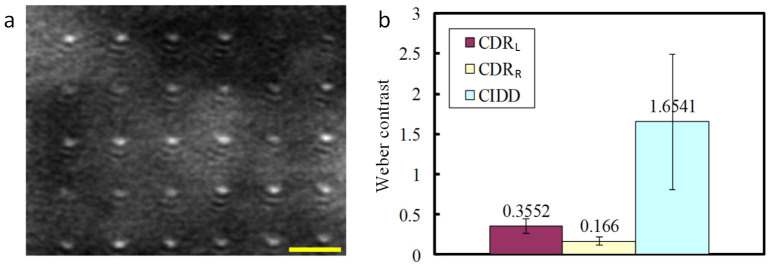
Imaging PCN's through tissue phantom. (a) 2D en-face CIDD image of PCN's; (b) Weber contrast comparison between CIDD, CDR_L_, and CDR_R_.

The L-PCN's were then imaged through the tissue phantom, in which a 1-mm-thick 1% Lipofundin solution was used as the scattering medium. Scattering apparently resulted in some speckle like scattering background that was, however, well below the CIDD signal from PCN's (Figure 6a). Individual nanoparticles were clearly visible. The background level was higher in CDR_L_ and CDR_R_ images, resulting in Weber contrasts of about one order of magnitude smaller than that of CIDD (Figure 6b). As expected, no nanostructures could be identified in conventional OCM and CIDS images of the same sample.

## Discussion

There is a strong need for imaging probes that can provide adequate contrasts with OCT/OCM. Such probes can be useful in high-sensitivity molecular imaging in highly scattering biological tissues. Conventional probes suffer from poor contrast and low sensitivity, as it is very often too challenging to effectively differentiate the probe signal from the background.

The ultra-high sensitivity provided by 3D chiral nanostructures is achieved by several contrast enhancing mechanisms. First of all, it is reasonable to assume that most of the backscattering/reflection from layered structure and symmetric scatterers are mostly co-polarized. By detecting the de-polarized component, most of the background is strongly suppressed. This is the reason that CDR is also an effective OCT/OCM contrast parameters when asymmetric nanostructures (including nanorods) are involved. Secondly, the residual background in depolarized signal is further reduced by the use of chiral structure and CIDD. In tissue samples it is expected that a certain degree of asymmetry in cellular and sub-cellular structures may still give rise to depolarized backscattering. However, as long as these structures are achiral, their contributions to CIDD are minimized. The Lipofundin solution in our tissue phantom contained essentially spherical scatterers. They did not lead to a strong depolarization background and there was a relatively weak background in CDR images. In case of OCT/OCM imaging in real tissues, the advantage of CIDD is likely to be more significant. Finally, plasmonic resonance significantly boosts the signal from the nanostructures. The experimentally demonstrated imaging depth of 1 mm is slightly lower than the typical OCT imaging depth. However, the backscattered signal from a single PCN is several orders of magnitude weaker than the reflectance from layered structures visualized by conventional OCT.

It has also been found in our experimental study that the commonly used chirality parameter, CIDS, does not provide a useful contrast for visualizing individual PCN's in a strong background. This is because co-polarized signals are overwhelmingly strong and that the difference between the left- and right-circularly polarized responses can be dominated by fluctuations caused by the imaging system and/or the sample (see the analysis following Figure S1 in the Supplementary Information).

The nanostructures used in this study were fabricated using e-beam lithography, which is time-consuming and costly. Chemical synthesis is more desirable for mass production of PCN's as OCT/OCM contrast agents. Recently a group has reported chemically synthesized 3D anisotropic gold nanorod dimer nanostructures^25^. It is expected that their method can be slightly modified to fabricate our design.

To extend our technique to in vivo tissue imaging and maintain the high sensitivity, more tissue characteristics have to be considered. The tissue phantom used in this study is essentially homogeneous. Most biological tissues, however, are heterogeneous and may induce sample aberrations. This is especially a problem in OCM, which uses a higher NA objective to reduce the spot size in the focal plane. The tightly focused illumination beam is desirable for higher spatial resolution as well as stronger backscattered signal from nanoparticles. Nevertheless, sample aberrations can greatly distort the focal distribution, resulting in diminished signal and enhanced background. It may be necessary to include adaptive optics in the OCM setup so that sample aberrations can be corrected or minimized.

## Methods

### Circular Polarization OCM image acquisition and processing

Our OCM setup was designed to obtain four circular polarization OCM image stacks in sequence. Each image stack consisted of 512 cross-sectional images for a specific combination of illumination polarization state and detection polarization selection. Limited by the scanning speed of the 2D scanning mirror (600 Hz typical), the acquisition speed for each cross-sectional OCM image was relatively slow. It took around 66 seconds to get one image stack and it took 264 seconds for a complete scan.

The raw OCM image stacks were processed using Matlab to reconstruct the en-face images in the focal plane. Due to uncompensated system and sample dispersion, OCM signal from a single object surface could be distributed to multiple en-face layers. These important layers were picked up and averaged to form a single en-face image. In addition, each en-face image was smoothed using a 3 × 3 filter operator to reduce the speckle noise.

### E-beam fabrication of nanostructures

Our nanostructures were fabricated at the Institute of Materials Research and Engineering (IMRE). In the fabrication process, ZEP resist was spin coated and developed on SiO_2_ substrate. E-beam evaporation was used to deposit Chromium base and an array of gold nanorods. Alignment markers were also deposited in this process. ZEP resist was removed by soaking the substrate into a remover. For the fabrication of PCN's, a dielectric SiO_2_ layer 50 nm in thickness was sputtered to cover the bottom layer nanorods. A second e-beam deposition process was then carried out to write the second layer of gold nanorods.

## Author Contributions

All authors (P.Z., K.M., S.R., and N.C.) contributed to writing of the manuscript. N.C. conceived the original idea. M.K. conducted FDTD numerical simulation. K.M. and P.Z. set up the CP-OCM system and carried out imaging experiments. P.Z. and K.M. performed image and data analysis with the assistance of N.C. and S.R.

## Supplementary Material

Supplementary Information Supplementary Information
