# Supplementary material for: Imaging single chiral nanoparticles in turbid media using circular-polarization optical coherence microscopy
Source: Sci Rep. 2014 May 15;4:4979. doi: 10.1038/srep04979 (PMC4021320; doi:10.1038/srep04979)
Supplement: Supplementary Information [file srep04979-s1.pdf]

Imaging single chiral nanoparticles in turbid media using circular-polarization optical coherence microscopy

Pengfei Zhang [1†], Kalpesh Mehta[2†] , Shakil Rehman[1,3], and Nanguang Chen[1,2]

[†]Equal Contributing Authors

[1]Optical Bioimaging Lab, Department of Bioengineering, National University of Singapore, 7 Engineering Drive 1, Singapore 117576

[2]NUS Graduate School for Integrative Sciences and Engineering, National University of Singapore, 28 Medical Drive, Singapore 117456

[3]BioSystems and Micromechanics IRG, Singapore MIT Alliance for Research and Technology, 1 Create Way, Singapore 138602

Correspondence and requests for materials should be addressed to N. C. (email: biecng@nus.edu.sg)

Supplementary Information

FDTD Simulation for Fabricated Plasmonic Chiral Nanostructure

FDTD simulations were performed to evaluate the polarization dependent responses of fabricated plasmonic chiral nanostructure according to its design dimensions. Figure S1a shows the cross-polarized components of the differential scattering cross-section (DSC) of a L-PCN for LCP (blue) and RCP (red) illuminations, respectively, as functions of wavelength. There is a significant difference between the two curves, which reaches its maximum around 920 nm. The co-polarized DSC's of the same structure are plotted in Figure S1b. The two spectra are fairly close to each other.

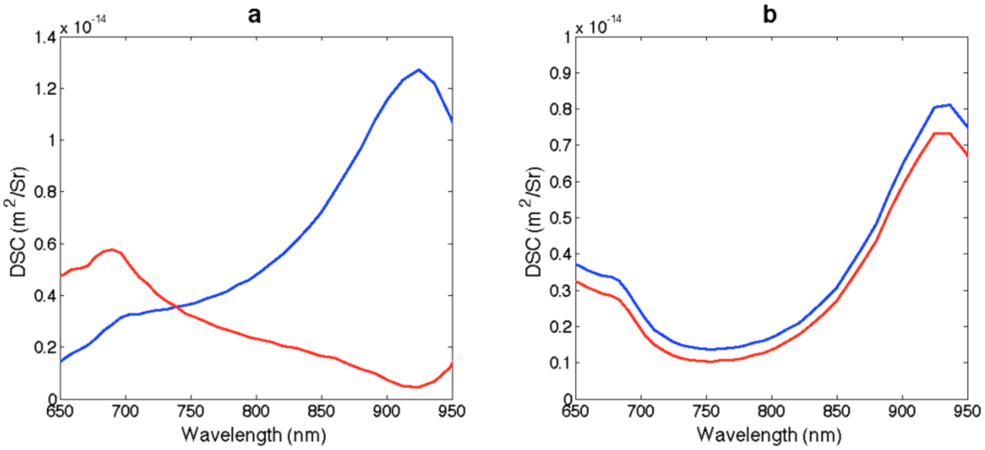

Figure S1: Backscattering spectra for L-PCN. (a) Cross-polarized DSC's for LCP (blue) and RCP (Red) illuminations; (b) Co-polarized DSC's for LCP (blue) and RCP (Red) illuminations.

From the simulated spectra, the CIDS and CIDD for an isolated L-PCN are estimated to be 0.382 and 1.816, respectively. When such a nanostructure is situated around a reflective surface (reflectivity: 0.04), the CIDS will become $1.44\times10^{-5}\pm5.24\times10^{-6}$ in the particle region and $0\pm5.24\times10^{-6}$ in the background region. The shot noise in the background reflectance is responsible for the fluctuations, which makes it difficult to differentiate the particle from the background. In the same scenario, the CIDD in the particle region will become $3.15\times10^{-5}\pm5.73\times10^{-8}$, which contains a negligible fluctuation. The CIDD background remains to be 0 if the surface reflectivity is polarization independent. The advantage of CIDD over CIDS is quite obvious in such a situation. The shot noise is estimated based on the following assumptions. The OCM/OCT system provides an incident power of 1 mW on the

sample; the objective has a NA of 0.15 (for both illumination and detection); the detection integration time is 0.1 milliseconds and the detection efficiency is 100%.

SEM Images at different stages of fabrication

SEM images of nanostructures were taken at initial stages of PCN array fabrication in order to confirm the accuracy in dimensions control. Figure S2 shows two gold nanorods in the bottom layer. They have not been covered with a dielectric layer. The dimensions of each nanorod were estimated 140 nm long and 25 nm wide. Shown in Figure S3 is another SEM of the same sample after a layer of 50-nm-thick SiO_2 layer was deposited on top of the bottom nanorods array. The image quality was visually deteriorated and the sizes of nanorods appeared to be significantly increased (roughly 210 nm by 70 nm). We believe that such a change was due to the limited penetration depth of SEM.

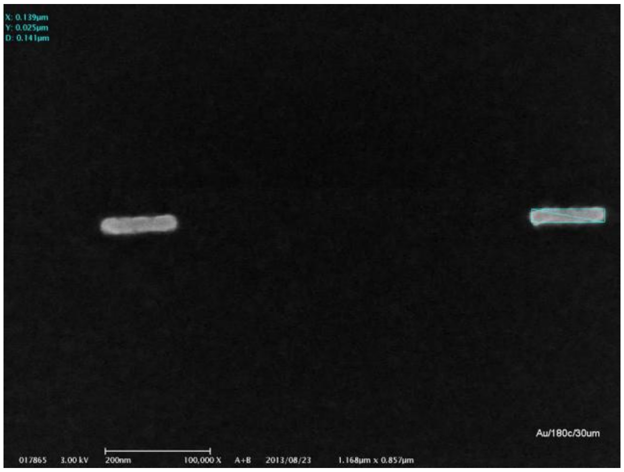

Figure S2: SEM image of gold nanorods patterned by E-beam.

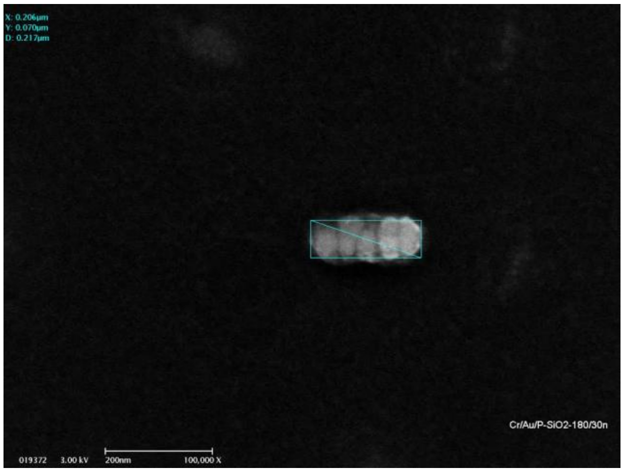

Figure S3: SEM image of a gold nanorod covered by a dielectric layer.

Monte Carlo simulation results predicting the degradation in the SEM resolution for subsurface structures

SEM has a rather limited penetration depth due to scattering of probing electrons by the sample. Monte Carlo simulation was performed to quantify the increased beam diameter once it entered a SiO_2 dielectric layer using a publically available Monte Carlo simulation tool:

http://nanonems.imb-cnm.csic.es/index.php?option=com_content&view=article&id=25%3Aeiss-electron-beam-monte-carlo-simulator&catid=2%3Ananofabrication&Itemid=9&lang=en

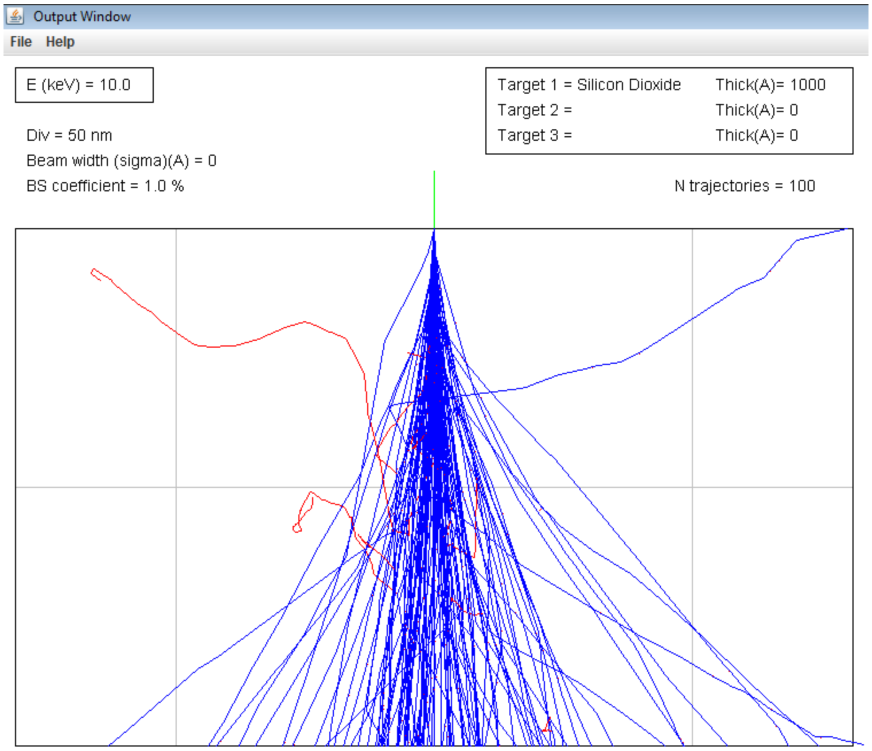

Figure S4: Electron beam propagation in SiO_2

Monte Carlo simulated electron trajectories are shown in the Figure S4. The beam diameter grows rapidly with an increase depth. The estimated degree of blurring is compatible with the imaging results (Figures S2 and S3).
